# Optimized selection of three major EGFR-TKIs in advanced EGFR-positive non-small cell lung cancer: a network metaanalysis

**DOI:** 10.18632/oncotarget.7713

**Published:** 2016-02-25

**Authors:** Yaxiong Zhang, Jin Sheng, Yunpeng Yang, Wenfeng Fang, Shiyang Kang, Yang He, Shaodong Hong, Jianhua Zhan, Yuanyuan Zhao, Cong Xue, Yuxiang Ma, Ting Zhou, Shuxiang Ma, Fangfang Gao, Tao Qin, Zhihuang Hu, Ying Tian, Xue Hou, Yan Huang, Ningning Zhou, Hongyun Zhao, Li Zhang

**Affiliations:** ^1^ Department of Medical Oncology, Sun Yat-sen University Cancer Center, Guangzhou, China; ^2^ State Key Laboratory of Oncology in South China, Guangzhou, China; ^3^ Collaborative Innovation Center for Cancer Medicine, Guangzhou, China; ^4^ Department of Anesthesiology, Sun Yat-sen University Cancer Center, Guangzhou, China; ^5^ Lau Luen Hung Private Medical Center, the First Affiliated Hospital of Sun Yat-sen University, Guangzhou, China

**Keywords:** EGFR-TKI, NSCLC, gefitinib, erlotinib, afatinib

## Abstract

**Background:** To answer which epidermal growth factor receptor-tyrosine kinase inhibitor (EGFR-TKI) is the best choice for advanced non-small cell lung cancer (NSCLC) EGFR mutants.

**Results:** 16 phase III randomized trials involving 2962 advanced NSCLC EGFR mutants were enrolled. Multiple treatment comparisons showed different EGFR-TKIs shared equivalent curative effect in terms of all outcome measures among the overall, chemo-naïve and previously treated patients. Rank probabilities showed that erlotinib and afatinib had potentially better efficacy compared with gefitinib in both of the overall and chemo-naïve patients. Potentially survival benefit of erlotinib was also observed in previously treated patients compared with gefitinib. Additionally, EGFR-TKI showed numerically greater survival benefit in 19 Del compared with chemotherapy, while it was opposite in 21 L858R. Furthermore, afatinib, erlotinib and gefitinib had high, moderate and low risk of rash & diarrhea, respectively, while the occurrence of elevated liver transaminase was more common in gefitinib.

**Methods:** Data of objective response rate (ORR), disease control rate (DCR), progression-free survival (PFS), overall survival (OS) and adverse events were extracted from included studies. Efficacy and toxicity of all included treatments were integrated by network meta-analyses.

**Conclusion:** Our study indicated a high efficacy-high toxicity pattern of afatinib, a high efficacy-moderate toxicity pattern of erlotinib and a medium efficacy-moderate toxicity pattern of gefitinib. Recommended EGFR-TKI should be suggested according to patients' tolerability and therapeutic efficacy in clinical practice. Moreover, the treatment for advanced EGFR-positive NSCLC might be different between 19 Del and 21 L858R.

## INTRODUCTION

It has been proved that three major epidermal growth factor receptor-tyrosine kinase inhibitors (EGFR-TKIs - gefitinib, erlotinib and afatinib) were the best choice for advanced chemo-naïve non-small cell lung cancer (NSCLC) patients harboring sensitive EGFR mutations from nine phase III, randomized trials. [1–9] Interestingly, the combined analyses of LUX-Lung 3 and LUX-Lung 6 showed that only patients with EGFR exon 19 deletions (19 Del) got overall survival benefit from afatinib compared with chemotherapy. By contrast, there were no significant differences between afatinib and chemotherapy in terms of survival for patients with EGFR L858R substitution in exon 21 (21 L858R). [10] After that, more and more studies focused on the subtypes of sensitive EGFR mutations, 19 Del and 21 L858R. Several meta-analyses proved that NSCLC patients with 19 Del had higher response rates and longer progression-free survival (PFS), overall survival (OS) after EGFR-TKI therapy compared with L858R. [11, 12] Moreover, a recent study found that NSCLC patients with 19 Del are more likely to be young and have lymphatic metastasis than those with L858R. [13] These findings suggest that EGFR 19 Del disease might be distinct from 21 L858R disease. Subgroups of 19 Del and L858R should be analyzed separately for therapeutic efficacy and toxicity in future researches.

With those different EGFR-TKIs, scientists performed some head-to-head randomized controlled trials (RCTs) to compare the efficacy and toxicity between gefitinib and erlotinib in chemo-naïve or previously treated patients. However, there was no statistically significant difference in response rates, PFS and OS for EGFR-mutated NSCLC. [14, 15] Additionally, a latest blockbuster trial which directly compared the first generation TKI with second generation TKI found that afatinib significantly improved the response rates and PFS in EGFR-mutant NSCLC versus gefitinib. Subgroup analyses found consistent effects in 19 Del patients and L858R patients. [16]

Up to now, we have sufficient clinical data of EGFR-TKIs in EGFR-positive NSCLC patients (EGFR mutants, 19 Del or 21 L858R cases). It is high time for us perform a large-scale analysis to answer which EGFR-TKI is the best clinical choice for EGFR-positive patients, 19 Del patients or L858R patients. Besides, we can analyze whether the optimized selection of EGFR-TKIs is different between chemo-naïve patients and previously treated ones. Since a single trial or conventional direct meta-analysis usually compares only two drugs, it is impossible to integrate information on the relative efficacy and toxicity of all optional regimens for the same indication. Therefore, a network meta-analysis which synthesizes data from both direct and indirect comparisons of diverse regimens is a superexcellent method to compare different treatments due to its good agreement on the real-world situation. [17] This efficacy and toxicity based network meta-analysis will help clinicians make precise choice of EGFR-TKI for advanced NSCLC EGFR mutants.

## RESULTS

### Eligible studies

**Figure 1:**
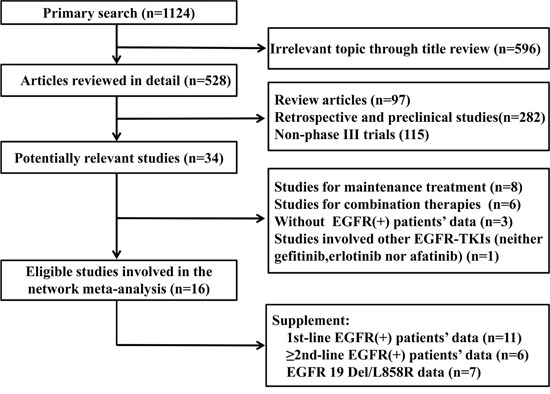
Profile summarizing the trial flow Abbreviations:19 Del, exon 19 deletion; 21 L858R, exon 21 L858R mutation.

1124 records were identified according to the primary search strategy and finally 16 phase III randomized trials were enrolled, [1-9, 14-16, 18-21] which involved 2962 advanced NSCLC patients with EGFR mutations. Figure 1 summarizes the flow chart. 11 trials focused on front-line therapy in 2531 treatment-naive patients, [1-9, 15, 16] while 6 trials investigated subsequent treatment after failure of chemotherapy in 522 previously treated patients. [18–21] In particular, we took the overall results of CTONG0901 as first-line trial outcomes because most of patients in CTONG0901 trial were chemo-naïve. The previously treated patients in CTONG0901 were incorporated into ≥ 2nd-line treatment group separately. [15] Table 1 summarized the basic characteristics of involved studies for this network meta-analysis. Table S1-S3 summarized the data of treatment efficacy (ORR, DCR, 1y-PFS rate, 1y-OS rate and 2y-OS rate) in EGFR mutants, 19 Del patients (N=1142) and 21 L858R ones (N=872). Table S4 enumerated 3 major adverse effects of EGFR-TKIs (rash, diarrhea and elevated LT) from eligible trials.

**Table 1:** Characteristics of included studies for meta-analyses

Trial (year)	Type	Race	Drug	EGFR mutants
IPASS	CT naive	Asian	Gefitinib	132
(2009)			TC	129
NEJ002	CT naive	Asian	Gefitinib	114
(2010)			TC	114
WJTOG3405	CT naive	Asian	Gefitinib	86
(2010)			DP	86
OPTIMAL	CT naive	Asian	Erlotinib	82
(2011)			GC	72
First-SIGNAL	CT naive	Asian	Gefitinib	26
(2012)			GC	16
EURTAC	CT naive	Caucasian	Erlotinib	86
(2012)			GP/DP/GC/DC	87
LUX-Lung 3	CT naive	Mixed	Afatinib	230
(2013)			AP	115
LUX-Lung 6	CT naive	Asian	Afatinib	242
(2014)			GP	122
ENSURE	CT naive	Asian	Erlotinib	110
(2015)			GP	107
LUX-Lung 7	CT naive	Mixed	Afatinib	160
(2015)			Gefitinib	159
V-15-32	Previously treated	Asian	Gefitinib	9
(2008)			DOC	11
INTEREST	Previously treated	Mixed	Gefitinib	19
(2008)			DOC	19
TITAN	Previously treated	Mixed	Erlotinib	7
(2012)			PEM/DOC	4
DELTA	Previously treated	Caucasian	Erlotinib	21
(2014)			DOC	30
WJOG 5108L	Previously treated	Asian	Gefitinib	161
(2014)			Erlotinib	150
CTONG 0901	Mixed(mainly on CT naïve) naïve)	Asian	Gefitinib	128
(2015)			Erlotinib	128
CTONG 0901	Previously treated	Asian	Gefitinib	44
(2015)			Erlotinib	47

Abbreviations: TC, carboplatin plus palitaxel; GP, cisplatin plus gemcitabine; DP, cisplatin plus docetaxel; DC, carboplatin plus docetaxel; DOC, docetaxel; GC, carboplatin plus gemcitabine; CT, chemotherapy; PEM, pemetrexed; AP, cisplatin plus pemetrexed; NA, not available.

### Single-arm meta-analyses on efficacy and toxicity of EGFR-TKI

Table 2 calculated the pooled efficacy and toxicity of EGFR-TKIs, gefitinib, erlotinib and afatinib in EGFR mutants or patients with 19 Del or 21 L858R. Only tiny numerical differences were observed among gefitinib, erlotinib and afatinib in terms of therapeutic efficacy in the whole population of EGFR mutants, as well as in chemo-naïve patients (both 19 Del and L858R). However, in previously treated patients, erlotinib showed better numerical data on 1y-PFS rate (**0.50**, 0.37-0.63), 1y-OS rate (**0.79**, 0.68-0.87) and 2y-OS rate (**0.57**, 0.37-0.76) than gefitinib (1y-PFS, **0.31**, 0.19-0.46; 1y-OS rate, **0.59**, 0.46-0.70; 2y-OS rate **0.30**, 0.20-0.43) (the efficacy of afatinib was not measured in previously treated patients). Particularly, in general, 19 Del patients had superior numerical data on treatment efficacy especially in ORR (**0.77**, 0.71-0.82), 1y-PFS rate (**0.45**, 0.40-0.50), 1y-OS rate (**0.87**, 0.83-0.91) and 2y-OS rate (**0.62**, 0.57-0.68) compared with 21 L858R patients (ORR, **0.60**, 0.52-0.67; 1y-PFS, **0.38**, 0.33-0.44; 1y-OS rate, **0.80**, 0.70-0.87; 2y-OS rate **0.47**, 0.34-0.60). Adverse effects were analyzed in treatment-naïve patients. Afatinib showed higher risk of rash (**0.86**, 0.80-0.91) (grade 3-4 rash, **0.14**, 0.10-0.18) and diarrhea (**0.91**, 0.86-0.95) (grade 3-4 diarrhea, **0.10**, 0.06-0.17) than gefitinib or erlotinib, while elevated LT (grade 3-4 elevated LT) was easily occurred in gefitinib (**0.36**, 0.15-0.65; **0.14**, 0.06-0.30) compared with afatinib or erlotinib.

**Table 2:** single-arm meta-analyses on efficacy and toxicity of EGFR-TKIs, gefitinib, erlotinib and afatinib in EGFR mutants or patients with 19 Del / 21 L858R

Efficacy / Toxicitiy	EGFR-TKIs	Gefitinib	Erlotinib	Afatinib
**EGFR mutants (Total)**				
**ORR**	0.65(0.61,0.69)	0.64(0.57,0.70)	0.66(0.57,0.74)	0.66(0.56,0.75)
**DCR**	0.90(0.88,0.92)	0.88(0.85,0.91)	0.90(0.86,0.92)	0.94(0.89,0.96)
**1y-PFS**	0.44(0.40,0.49)	0.40(0.36,0.43)	0.47(0.37,0.57)	0.52(0.47,0.57)
**1y-OS**	0.80(0.77,0.83)	0.78(0.71,0.84)	0.80(0.75,0.85)	0.82(0.78,0.85)
**2y-OS**	0.51(0.46,0.56)	0.50(0.40,0.60)	0.50(0.44,0.57)	0.52(0.38,0.65)
**EGFR mutants (First-line)**				
**ORR**	0.66(0.61,0.71)	0.66(0.57,0.74)	0.68(0.58,0.77)	0.66(0.56,0.75)
**DCR**	0.91(0.88,0.93)	0.89(0.85,0.92)	0.90(0.85,0.94)	0.94(0.89,0.96)
**1y-PFS**	0.45(0.40,0.50)	0.40(0.36,0.44)	0.47(0.34,0.61)	0.52(0.47,0.57)
**1y-OS**	0.80(0.78,0.83)	0.80(0.74,0.85)	0.80(0.74,0.85)	0.82(0.78,0.85)
**2y-OS**	0.51(0.46,0.56)	0.53(0.43,0.62)	0.49(0.44,0.54)	0.52(0.38,0.65)
**EGFR mutants (After first-line)**				
**ORR**	0.60(0.53,0.66)	0.59(0.45,0.72)	0.58(0.50,0.66)	
**DCR**	0.87(0.83,0.91)	0.86(0.79,0.91)	0.89(0.82,0.93)	
**1y-PFS**	0.41(0.32,0.51)	0.31(0.19,0.46)	0.50(0.37,0.63)	
**1y-OS**	0.70(0.57,0.81)	0.59(0.46,0.70)	0.79(0.68,0.87)	
**2y-OS**	0.45(0.29,0.62)	0.30(0.20,0.43)	0.57(0.37,0.76)	
**EGFR 19 Del (First-line)**				
**ORR**	0.77(0.71,0.82)	0.81(0.72,0.87)	0.78(0.64,0.89)	0.74(0.64,0.83)
**DCR**	0.96(0.91,0.98)	1.00(0.96,1.00)	0.98(0.88,1.00)	0.94(0.88,0.97)
**1y-PFS**	0.45(0.40,0.50)	0.42(0.35,0.49)	0.46(0.37,0.55)	0.51(0.40,0.61)
**1y-OS**	0.87(0.83,0.91)	NA	0.86(0.74,0.94)	0.88(0.83,0.91)
**2y-OS**	0.62(0.57,0.68)	NA	0.60(0.46,0.72)	0.63(0.56,0.70)
**EGFR 21 L858R (First-line)**				
**ORR**	0.60(0.52,0.67)	0.56(0.46,0.65)	0.73(0.50,0.89)	0.60(0.48,0.72)
**DCR**	0.93(0.89,0.95)	0.94(0.85,0.98)	0.95(0.77,1.00)	0.93(0.86,0.97)
**1y-PFS**	0.38(0.33,0.44)	0.39(0.32,0.47)	0.32(0.23,0.43)	0.42(0.30,0.54)
**1y-OS**	0.80(0.70,0.87)	NA	0.83(0.70,0.92)	0.79(0.63,0.89)
**2y-OS**	0.47(0.34,0.60)	NA	0.52(0.38,0.66)	0.45(0.26,0.65)
**EGFR mutants (First-line)**				
**Rash**	0.78(0.73,0.83)	0.75(0.64,0.84)	0.73(0.68,0.77)	0.86(0.80,0.91)
**Diarrhea**	0.57(0.37,0.75)	0.41(0.23,0.61)	0.35(0.19,0.55)	0.91(0.86,0.95)
**Elevated LT**	0.20(0.11,0.34)	0.36(0.15,0.65)	0.12(0.04,0.31)	0.14(0.06,0.28)
**Grade 3-4 Rash**	0.06(0.04,0.10)	0.03(0.02,0.06)	0.05(0.02,0.12)	0.14(0.10,0.18)
**Grade 3-4 Diarrhea**	0.04(0.02,0.07)	0.01(0.00,0.02)	0.02(0.00,0.10)	0.10(0.06,0.17)
**Grade 3-4 Elevated LT**	0.04(0.02,0.10)	0.14(0.06,0.30)	0.02(0.01,0.06)	0.01(0.00,0.04)

Abbreviations:19 Del, exon 19 deletion; 21 L858R, exon 21 L858R mutation; ORR, objective response rate; DCR, disease control rate; PFS, progression-free survival; OS, overall survival; LT, liver transaminase, NA, not available.

### Networks for multiple treatment comparisons (MTC)

**Figure 2:**
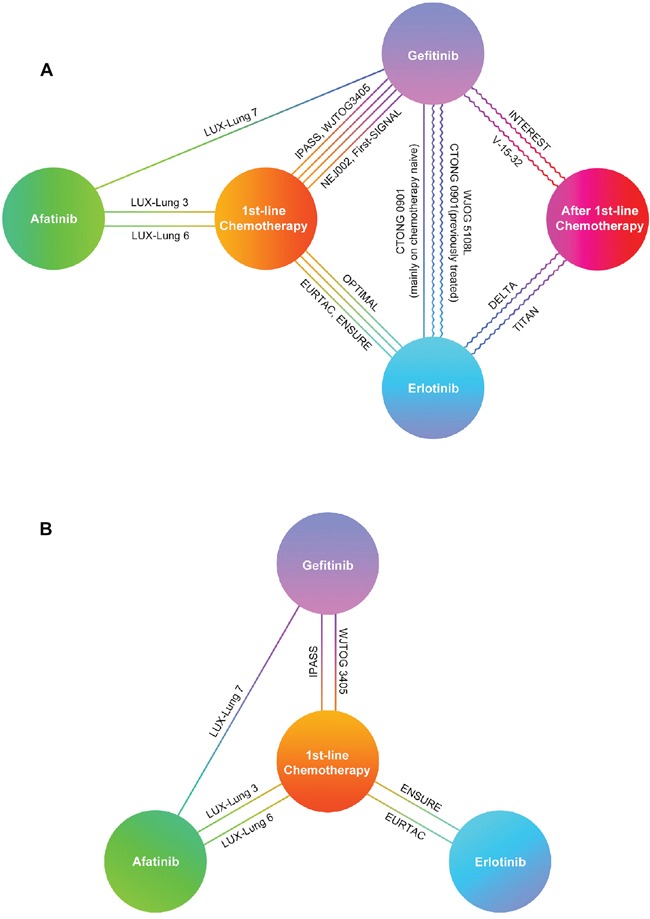
Network established for multiple treatment comparisons **A.** For EGFR mutants; **B.** For patients with EGFR 19 Del or 21 L858R. Straight lines between drugs represented comparisons in chemotherapy-naïve patients. Curve lines between drugs represented comparisons in previously treated patients. Abbreviations:19 Del, exon 19 deletion; 21 L858R, exon 21 L858R mutation.

Network A and B was established for MTC based on available data of outcomes in EGFR mutants and patients with EGFR 19 Del / 21 L858R, respectively (Figure 2). It was important to note that network A was composed of first-line trials and ≥ 2nd-line trials so that it could be divided into two sub-networks for MTC in chemo-naïve patients or previously treated patients.

### Network meta-analyses on therapeutic efficacy in overall & chemo-naïve EGFR mutants

**Figure 3:**
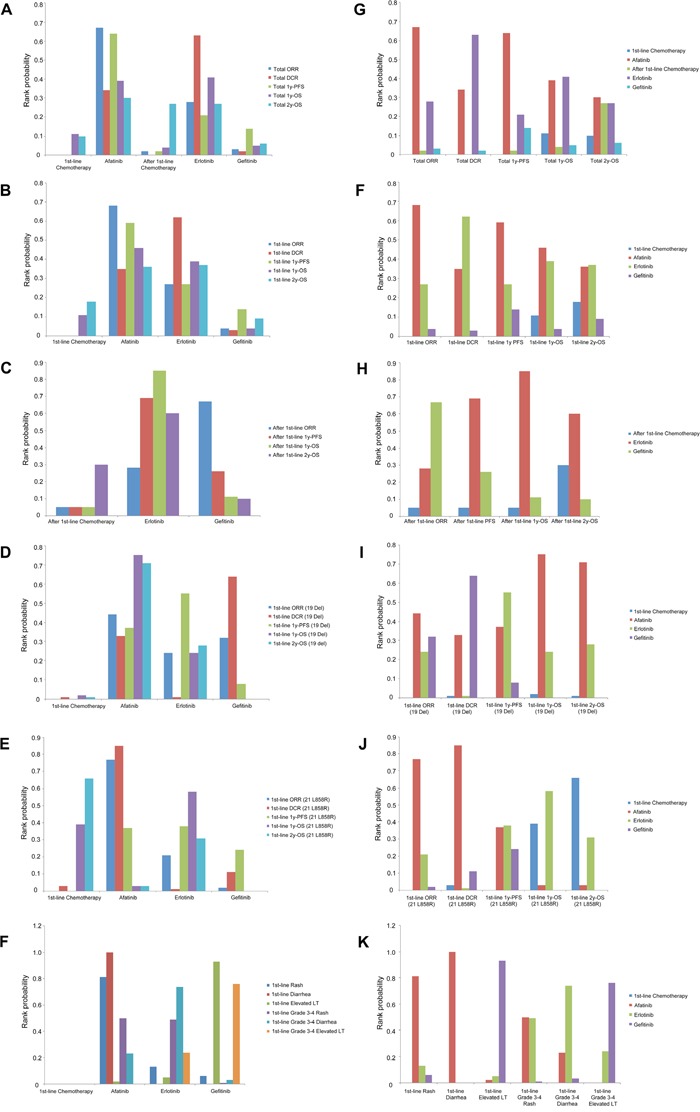
Distribution of probabilities of each agent being ranked the first place based on network A and network B A, B, C, D, E, F were classified by drugs; G, H, I, J, K, L were classified by outcomes. Abbreviations:19 Del, exon 19 deletion; 21 L858R, exon 21 L858R mutation; ORR, objective response rate; DCR, disease control rate; PFS, progression-free survival; OS, overall survival; LT, liver transaminase.

According to the data based on network A, gefitinib, elotinib and afatinib shared equivalent curative effect in all outcome measures without significant differences in ORs while all EGFR-TKIs were better than first-line chemotherapy in terms of ORR, DCR and 1y-PFS rate. No significant difference of OS among all TKIs and chemotherapy (Table 3). Analyses of the probability to be the best treatment indicated that afatinib ranked best among all the TKIs in terms of ORR (overall 0.67; chemo-naïve 0.68) and 1-year PFS (overall 0.64; chemo-naïve 0.59), while erlotinib ranked best for DCR (overall 0.63; chemo-naïve 0.62). Erlotinib and afatinib shared similar superior rankings with respect to 1y-OS (overall 0.41 and 0.39; chemo-naïve 0.39 and 0.46) and 2y-OS (overall 0.27 and 0.30; chemo-naïve 0.37 and 0.36) compared with gefitinib (Figure 3A, 3B, 3G, 3F and Table S5).

**Table 3:** Multiple treatment comparison for efficacy and toxicities based on network A and network B

**Total ORR (EGFR mutants)**				
**1st-line Chemotherapy**	6.08 (3.55, 10.89)	1.55 (0.37, 6.10)	5.07 (3.20, 8.74)	4.22 (2.80, 6.69)
0.16 (0.09, 0.28)	**Afatinib**	0.26 (0.06, 1.07)	0.84 (0.42, 1.75)	0.69 (0.38, 1.28)
0.64 (0.16, 2.69)	3.92 (0.94, 17.13)	**After 1st-line Chemotherapy**	3.26 (0.82, 14.04)	2.71 (0.76, 10.51)
0.20 (0.11, 0.31)	1.20 (0.57, 2.39)	0.31 (0.07, 1.22)	**Erlotinib**	0.84 (0.49, 1.35)
0.24 (0.15, 0.36)	1.44 (0.78, 2.62)	0.37 (0.10, 1.31)	1.19 (0.74, 2.05)	**Gefitinib**
**Total DCR (EGFR mutants)**				
**1st-line Chemotherapy**	2.36 (1.30, 4.21)	2.75 (1.56, 4.52)	1.83 (1.12, 2.94)	
0.42 (0.24, 0.77)	**Afatinib**	1.13 (0.52, 2.39)	0.78 (0.38, 1.47)	
0.36 (0.22, 0.64)	0.88 (0.42, 1.94)	**Erlotinib**	0.68 (0.41, 1.12)	
0.55 (0.34, 0.89)	1.28 (0.68, 2.66)	1.46 (0.89, 2.46)	**Gefitinib**	
**Total 1y-PFS (EGFR mutants)**				
**1st-line Chemotherapy**	8.33 (3.73, 20.48)	2.00 (0.37, 9.12)	6.42 (3.12, 12.86)	6.19 (3.35, 12.00)
0.12 (0.05, 0.27)	**Afatinib**	0.24 (0.04, 1.25)	0.77 (0.26, 2.09)	0.74 (0.29, 1.85)
0.50 (0.11, 2.69)	4.22 (0.80, 27.13)	**After 1st-line Chemotherapy**	3.25 (0.81, 14.64)	3.08 (0.71, 17.42)
0.16 (0.08, 0.32)	1.30 (0.48, 3.90)	0.31 (0.07, 1.23)	**Erlotinib**	0.96 (0.49, 2.14)
0.16 (0.08, 0.30)	1.35 (0.54, 3.41)	0.32 (0.06, 1.41)	1.04 (0.47, 2.05)	**Gefitinib**
**Total 1y-OS (EGFR mutants)**				
**1st-line Chemotherapy**	1.08 (0.64, 1.91)	0.48 (0.17, 1.53)	1.08 (0.69, 1.71)	0.84 (0.53, 1.29)
0.92 (0.52, 1.57)	**Afatinib**	0.44 (0.13, 1.51)	1.02 (0.48, 2.01)	0.77 (0.37, 1.51)
2.08 (0.65, 5.91)	2.28 (0.66, 7.52)	**After 1st-line Chemotherapy**	2.28 (0.79, 6.35)	1.72 (0.60, 4.65)
0.93 (0.59, 1.46)	0.98 (0.50, 2.07)	0.44 (0.16, 1.27)	**Erlotinib**	0.76 (0.45, 1.30)
1.19 (0.78, 1.88)	1.30 (0.66, 2.70)	0.58 (0.21, 1.68)	1.31 (0.77, 2.24)	**Gefitinib**
**Total 2y-OS (EGFR mutants)**				
**1st-line Chemotherapy**	1.03 (0.69, 1.52)	0.87 (0.33, 2.16)	1.04 (0.74, 1.43)	0.91 (0.66, 1.24)
0.97 (0.66, 1.45)	**Afatinib**	0.84 (0.31, 2.20)	1.02 (0.60, 1.66)	0.89 (0.54, 1.46)
1.15 (0.46, 3.04)	1.19 (0.45, 3.22)	**After 1st-line Chemotherapy**	1.19 (0.47, 2.95)	1.05 (0.41, 2.70)
0.96 (0.70, 1.36)	0.98 (0.60, 1.67)	0.84 (0.34, 2.11)	**Erlotinib**	0.87 (0.62, 1.31)
1.10 (0.81, 1.51)	1.12 (0.68, 1.85)	0.95 (0.37, 2.41)	1.15 (0.76, 1.62)	**Gefitinib**
**1st-line ORR (EGFR mutants)**				
**1st-line Chemotherapy**	6.08 (3.47, 11.25)	5.15 (3.22, 8.98)	4.25 (2.81, 7.00)	
0.16 (0.09, 0.29)	**Afatinib**	0.84 (0.42, 1.80)	0.70 (0.37, 1.35)	
0.19 (0.11, 0.31)	1.19 (0.55, 2.41)	**Erlotinib**	0.83 (0.50, 1.39)	
0.24 (0.14, 0.36)	1.44 (0.74, 2.68)	1.21 (0.72, 2.02)	**Gefitinib**	
**1st-line DCR (EGFR mutants)**				
**1st-line Chemotherapy**	2.37 (1.27, 4.31)	2.71 (1.54, 5.14)	1.79 (1.07, 3.12)	
0.42 (0.23, 0.79)	**Afatinib**	1.15 (0.53, 2.82)	0.76 (0.39, 1.55)	
0.37 (0.19, 0.65)	0.87 (0.35, 1.90)	**Erlotinib**	0.65 (0.33, 1.25)	
0.56 (0.32, 0.93)	1.32 (0.65, 2.55)	1.53 (0.80, 3.02)	**Gefitinib**	
**1st-line 1y-PFS (EGFR mutants)**				
**1st-line Chemotherapy**	8.31 (3.19, 23.21)	6.57 (2.74, 15.10)	6.12 (2.87, 13.50)	
0.12 (0.04, 0.31)	**Afatinib**	0.78 (0.21, 2.69)	0.73 (0.25, 2.09)	
0.15 (0.07, 0.37)	1.28 (0.37, 4.74)	**Erlotinib**	0.93 (0.36, 2.65)	
0.16 (0.07, 0.35)	1.37 (0.48, 3.96)	1.07 (0.38, 2.79)	**Gefitinib**	
**1st-line 1y-OS (EGFR mutants)**				
**1st-line Chemotherapy**	1.11 (0.62, 1.96)	1.08 (0.67, 1.72)	0.80 (0.50, 1.27)	
0.90 (0.51, 1.61)	**Afatinib**	0.98 (0.46, 2.09)	0.72 (0.35, 1.55)	
0.92 (0.58, 1.50)	1.02 (0.48, 2.17)	**Erlotinib**	0.74 (0.42, 1.29)	
1.25 (0.79, 1.99)	1.39 (0.65, 2.86)	1.35 (0.78, 2.37)	**Gefitinib**	
**1st-line 2y-OS (EGFR mutants)**				
**1st-line Chemotherapy**	1.00 (0.70, 1.46)	1.02 (0.72, 1.43)	0.91 (0.66, 1.23)	
1.00 (0.68, 1.43)	**Afatinib**	1.01 (0.60, 1.64)	0.91 (0.55, 1.47)	
0.98 (0.70, 1.38)	0.99 (0.61, 1.66)	**Erlotinib**	0.89 (0.61, 1.34)	
1.10 (0.81, 1.51)	1.10 (0.68, 1.82)	1.13 (0.75, 1.63)	**Gefitinib**	
**After 1st-line ORR (EGFR mutants)**				
**After 1st-line Chemotherapy**	2.22 (0.36, 12.79)	2.83 (0.71, 11.29)		
0.45 (0.08, 2.75)	**Erlotinib**	1.29 (0.40, 4.13)		
0.35 (0.09, 1.42)	0.77 (0.24, 2.52)	**Gefitinib**		
**After 1st-line 1y-PFS (EGFR mutants)**				
**After 1st-line Chemotherapy**	3.63 (0.66, 25.65)	2.61 (0.37, 27.85)		
0.28 (0.04, 1.51)	**Erlotinib**	0.72 (0.19, 3.16)		
0.38 (0.04, 2.68)	1.38 (0.32, 5.19)	**Gefitinib**		
**After 1st-line 1y-OS (EGFR mutants)**				
**After 1st-line Chemotherapy**	2.65 (0.76, 9.46)	1.42 (0.39, 4.88)		
0.38 (0.11, 1.31)	**Erlotinib**	0.52 (0.18, 1.62)		
0.70 (0.20, 2.58)	1.92 (0.62, 5.51)	**Gefitinib**		
**After 1st-line 2y-OS (EGFR mutants)**				
**After 1st-line Chemotherapy**	1.29 (0.34, 4.55)	0.72 (0.17, 2.97)		
0.78 (0.22, 2.92)	**Erlotinib**	0.57 (0.17, 2.07)		
1.38 (0.34, 5.72)	1.76 (0.48, 6.02)	**Gefitinib**		
**1st-line ORR (19 Del)**				
**1st-line Chemotherapy**	8.32 (2.76, 25.95)	5.32 (0.86, 32.59)	7.37 (1.85, 27.80)	
0.12 (0.04, 0.36)	**Afatinib**	0.65 (0.08, 5.44)	0.89 (0.22, 3.48)	
0.19 (0.03, 1.16)	1.53 (0.18, 13.17)	**Erlotinib**	1.39 (0.14, 13.80)	
0.14 (0.04, 0.54)	1.12 (0.29, 4.48)	0.72 (0.07, 6.96)	**Gefitinib**	
**1st-line DCR (19 Del)**				
**1st-line Chemotherapy**	3.53 (0.78, 15.40)	0.00 (0.00, 3.73)	565.06 (0.00, 3E16)	
0.28 (0.06, 1.28)	**Afatinib**	0.00 (0.00, 1.26)	177.99 (0.00, 8E15)	
2E3 (0.27, 9E13)	9E3 (0.79, 3E14)	**Erlotinib**	7E6 (0.00, 3E23)	
0.00 (0.00, 9E12)	0.01 (0.00, 3E13)	0.00 (0.00, 6E9)	**Gefitinib**	
**1st-line 1y-PFS (19 Del)**				
**1st-line Chemotherapy**	10.47 (0.44, 242.61)	13.76 (2.21, 112.29)	7.01 (1.11, 46.12)	
0.10 (0.00, 2.25)	**Afatinib**	1.32 (0.03, 58.35)	0.71 (0.05, 8.28)	
0.07 (0.01, 0.45)	0.76 (0.02, 32.80)	**Erlotinib**	0.50 (0.03, 7.16)	
0.14 (0.02, 0.90)	1.41 (0.12, 18.20)	1.99 (0.14, 30.48)	**Gefitinib**	
**1st-line 1y-OS (19 Del)**				
**1st-line Chemotherapy**	1.98 (0.87, 4.52)	1.18 (0.33, 4.31)		
0.51 (0.22, 1.14)	**Afatinib**	0.60 (0.14, 2.79)		
0.85 (0.23, 3.00)	1.67 (0.36, 7.35)	**Erlotinib**		
**1st-line 2y-OS (19 Del)**				
**1st-line Chemotherapy**	2.02 (0.94, 4.28)	1.42 (0.45, 4.34)		
0.50 (0.23, 1.07)	**Afatinib**	0.70 (0.17, 2.75)		
0.70 (0.23, 2.21)	1.43 (0.36, 5.74)	**Erlotinib**		
**1st-line ORR (21 L858R)**				
**1st-line Chemotherapy**	4.44 (1.88, 11.36)	2.26 (0.47, 12.96)	1.49 (0.52, 4.62)	
0.22 (0.09, 0.53)	**Afatinib**	0.52 (0.08, 3.36)	0.33 (0.12, 0.98)	
0.44 (0.08, 2.13)	1.93 (0.30, 12.24)	**Erlotinib**	0.65 (0.09, 4.69)	
0.67 (0.22, 1.92)	2.99 (1.02, 8.69)	1.55 (0.21, 11.13)	**Gefitinib**	
**1st-line DCR (21 L858R)**				
**1st-line Chemotherapy**	3.54 (0.82, 16.07)	0.00 (0.00, 1.18)	0.68 (0.01, 16.19)	
0.28 (0.06, 1.22)	**Afatinib**	0.00 (0.00, 0.41)	0.19 (0.00, 3.20)	
5E5 (0.85, 2E18)	1E6 (2.44, 9E18)	**Erlotinib**	3E5 (0.16, 1E18)	
1.48 (0.06, 85.73)	5.25 (0.31, 206.14)	0.00 (0.00, 6.35)	**Gefitinib**	
**1st-line 1y-PFS (21 L858R)**				
**1st-line Chemotherapy**	5.09 (0.67, 46.45)	4.88 (1.24, 20.48)	4.88 (1.42, 19.92)	
0.20 (0.02, 1.50)	**Afatinib**	0.95 (0.07, 10.44)	0.98 (0.19, 4.74)	
0.20 (0.05, 0.81)	1.05 (0.10, 13.86)	**Erlotinib**	1.01 (0.15, 7.17)	
0.20 (0.05, 0.71)	1.02 (0.21, 5.40)	0.99 (0.14, 6.53)	**Gefitinib**	
**1st-line 1y-OS (21 L858R)**				
**1st-line Chemotherapy**	0.50 (0.21, 1.14)	1.15 (0.33, 4.53)		
1.98 (0.88, 4.79)	**Afatinib**	2.30 (0.52, 11.23)		
0.87 (0.22, 3.05)	0.44 (0.09, 1.93)	**Erlotinib**		
**1st-line 2y-OS (21 L858R)**				
**1st-line Chemotherapy**	0.50 (0.21, 1.15)	0.76 (0.22, 2.56)		
2.01 (0.87, 4.68)	**Afatinib**	1.52 (0.34, 6.62)		
1.32 (0.39, 4.55)	0.66 (0.15, 2.91)	**Erlotinib**		
**1st-line Rash (EGFR mutants)**				
**1st-line Chemotherapy**	62.76 (15.29, 284.27)	27.77 (8.24, 101.86)	24.42 (6.74, 92.46)	
0.02 (0.00, 0.07)	**Afatinib**	0.45 (0.07, 2.81)	0.39 (0.07, 2.09)	
0.04 (0.01, 0.12)	2.23 (0.36, 15.08)	**Erlotinib**	0.87 (0.19, 4.07)	
0.04 (0.01, 0.15)	2.57 (0.48, 13.61)	1.15 (0.25, 5.19)	**Gefitinib**	
**1st-line Diarrhea (EGFR mutants)**				
**1st-line Chemotherapy**	61.67 (20.69, 191.84)	6.44 (2.46, 17.02)	5.67 (2.19, 15.74)	
0.02 (0.01, 0.05)	**Afatinib**	0.10 (0.03, 0.41)	0.09 (0.03, 0.32)	
0.16 (0.06, 0.41)	9.56 (2.41, 38.61)	**Erlotinib**	0.88 (0.27, 2.88)	
0.18 (0.06, 0.46)	10.84 (3.17, 36.09)	1.13 (0.35, 3.64)	**Gefitinib**	
**1st-line Elevated LT (EGFR mutants)**				
**1st-line Chemotherapy**	1.19 (0.43, 3.31)	1.64 (0.78, 3.70)	3.26 (1.59, 7.00)	
0.84 (0.30, 2.33)	**Afatinib**	1.36 (0.42, 4.92)	2.70 (1.03, 7.73)	
0.61 (0.27, 1.28)	0.74 (0.20, 2.40)	**Erlotinib**	2.01 (0.76, 5.04)	
0.31 (0.14, 0.63)	0.37 (0.13, 0.97)	0.50 (0.20, 1.32)	**Gefitinib**	
**1st-line Grade 3-4 Rash (EGFR mutants)**				
**1st-line Chemotherapy**	207.88 (7.29, 5E4)	198.02 (6.13, 8E4)	11.01 (0.46, 806.99)	
0.00 (0.00, 0.14)	**Afatinib**	0.96 (0.00, 434.91)	0.06 (0.00, 2.21)	
0.01 (0.00, 0.16)	1.04 (0.00, 386.86)	**Erlotinib**	0.06 (0.00, 4.85)	
0.09 (0.00, 2.16)	16.80 (0.45, 2E3)	16.46 (0.21, 8E3)	**Gefitinib**	
**1st-line Grade 3-4 Diarrhea (EGFR mutants)**				
**1st-line Chemotherapy**	2E6 (224.49, 2E15)	5E9 (67.39, 1E19)	1E5 (12.91, 2E14)	
0.00 (0.00, 0.00)	**Afatinib**	4E3 (0.00, 2E11)	0.08 (0.00, 8.85)	
0.00 (0.00, 0.01)	0.00 (0.00, 1E10)	**Erlotinib**	0.00 (0.00, 7E8)	
0.00 (0.00, 0.08)	12.77 (0.11, 1E3)	5E4 (0.00, 8E12)	**Gefitinib**	
**1st-line Grade 3-4 Elevated LT (EGFR mutants)**				
**1st-line Chemotherapy**	0.48 (0.01, 7.22)	8.37 (0.53, 1E3)	38.04 (4.38, 625.53)	
2.07 (0.14, 85.06)	**Afatinib**	18.94 (0.42, 1E4)	84.42 (4.94, 5E3)	
0.12 (0.00, 1.88)	0.05 (0.00, 2.38)	**Erlotinib**	4.38 (0.03, 182.97)	
0.03 (0.00, 0.23)	0.01 (0.00, 0.20)	0.23 (0.01, 37.33)	**Gefitinib**	

Abbreviations:19 Del, exon 19 deletion; 21 L858R, exon 21 L858R mutation; ORR, objective response rate; DCR, disease control rate; PFS, progression-free survival; OS, overall survival; LT, liver transaminase.

### Network meta-analyses on therapeutic efficacy in previously treated EGFR mutants

Only gefitinib, elotinib and chemotherapy did MTC based on sub-network (≥ 2nd-line trials) of network A. Above three treatments shared equivalent efficacy in all outcome measures by showing no significant differences in ORs (Table 3). Rank probabilities indicated gefitinib ranked best for ORR (0.67) while erlotinib ranked best for 1y-PFS (0.69), 1y-OS (0.85) and 2y-OS (0.60) compared with gefitinib and ≥ 2nd-line chemotherapy (Figure 3C, 3H and Table S5). DCRs were not evaluated in previously treated EGFR mutants due to not available data.

### Network meta-analyses on therapeutic efficacy in patients with EGFR 19 Del / 21 L858R

According to the data based on network B, gefitinib, elotinib and afatinib showed similar curative effect in all outcome measures without significant differences in ORs in EGFR 19 Del patients, while afatinib had better ORR than gefitinib and higher DCR than erlotinib in EGFR 21 L858R patients. EGFR-TKI showed numerically greater survival benefit in 19 Del compared with chemotherapy, while it was opposite in 21 L858R (Table 3). Gefitinib was not measured in 19 Del / 21 L858R for OS because of lack of available data. Rank probabilities revealed that EGFR-TKIs ranked best for ORR, DCR and 1y-PFS compared with chemotherapy in 19 Del patients, as well as 21 L858R patients. For 1y-OS (afatinib 0.75, erlotinib 0.24) and 2y-OS (afatinib 0.71, erlotinib 0.28), EGFR-TKIs still ranked best in 19 Del patients. Nevertheless, the superiority of EGFR-TKI in OS was reduced in 21 L858R patients for 1y-OS (chemotherapy 0.39, erlotinib 0.58). Furthermore, chemotherapy ranked best for 2y-OS (0.66) compared with EGFR-TKIs (Figure 3D, 3E, 3I, 3J and Table S5).

### Network meta-analyses on toxicity in EGFR mutants

The occurrence rates of rash, grade 3-4 rash and grade 3-4 diarrhea showed no significant differences among gefitinib, elotinib and afatinib. However, afatinib showed higher risk of diarrhea than gefitinib or erlotinib, while gefitinib had greater risk of elevated LT and grade 3-4 elevated LT compared with afatinib or erlotinib (Table 3). Rank probabilities showed that afatinib ranked first for the risk of rash (0.81) and diarrhea (1.00) and shared similar rank (0.50) with erlotinib (0.49) for the risk of grade 3-4 rash. Erlotinib ranked first for grade 3-4 diarrhea (0.74) compared with gefitinib and afatinib. Besides, gefitinib ranked first for the risk of elevated LT (0.93) and grade 3-4 elevated LT (0.76) compared with afatinib or erlotinib (Figure 3F, 3K and Table S5).

## DISCUSSION

### The origin of this network meta-analysis

A previous MTC tried to provide indirect comparison for available EGFR-TKIs in treating patients with advanced NSCLC who harbor EGFR mutations. [22] However, at that time, there was no direct head to head comparison between these agents. As a result, the entire network in previous meta-analysis was not well-established. Besides, the results of the previous study were short of stratifications by treatment lines or subtypes of EGFR mutations, which didn't reach to precise medical level. Therefore, we still need a large-scale network meta-analysis making multiple comparisons of currently available EGFR-TKIs urgently which integrates the latest data of head to head trials and performs sub-network meta-analyses by different treatment lines and EGFR mutant subtypes.

### Intellectual consideration of the established network

In order to ensure the reliability of research and the exchangeability of cross-treatment, only phase III randomized trials with strict patient allocation and optimized balance between treatment and control group were included in our study. Additionally, icotinib was not enrolled in the network because of following reasons [23]: (i) ICOGEN was the only phase III trial which focused on icotinib by far. It might break the stability of the network because each regimen of the network is encouraged to be linked with other treatments as much as possible. (ii) We still lack of sufficient data on the efficacy of icotinib compared with other EGFR-TKIs or chemotherapy. (iii) Icotinib was not approved in the international market except for China. The world-wide usage rate of icotinib was much lower than gefitinib or erlotinib. Besides, each regimen of the first-line or after first-line chemotherapy was platinum-based doublet or single agent chemotherapy, thus insuring concordant therapeutic efficacy within groups. Therefore, the consistency across the newly-established network would harmonize with real situation.

### MTC of efficacy in the overall, chemo-naïve and previously treated EGFR mutants

Our study showed that different EGFR-TKIs shared equivalent curative effect in terms of all outcome measures among the overall, chemo-naïve and previously treated EGFR mutants. EGFR-TKIs were better than first-line chemotherapy in terms of ORR, DCR and 1y-PFS rate, instead of OS. The superiority of EGFR-TKIs in ORR, DCR and PFS for EGFR mutants was due to block of EGFR-driven signals, while the failure to make a distinction for OS between EGFR-TKIs and chemotherapy could be explained by the influence of subsequent crossed treatments.

Rank probabilities showed that erlotinib and afatinib had potentially better efficacy compared with gefitinib in both of the overall and chemo-naïve EGFR mutants. Potentially survival benefit of erlotinib was also observed in previously treated patients compared with gefitinib. Previous trials showed that the reference dose of gefitinib (250 mg qd) was administered at approximately one third of its maximum-tolerated dose (MTD) while erlotinib (150 mg qd) and afatinib (40 mg qd) almost reached their MTDs, respectively. [24–26] Moreover, the half-maximal inhibitory concentration value of erlotinib was significant smaller than that of gefitinib. [27] As a result, the differences in biological dose of gefitinb, erlotinib and afatinib might be a possible reason for the above trends. Besides, afatinib, as a second-generation TKI, had the ability of irreversibly inhibiting EGFR-kinases and suppressing all ErbB receptor family,[28] which might show stronger efficacy than first-generation TKI due to its better binding strength with the EGFR and wider blockade of other signaling networks.

Based on the rank results, afatinib and erlotinib might be superior choices for chemo-naïve EGFR mutant patients as regards efficacy, while erlotinib showed its potentially survival benefit in previously treated patients. However, we still lack of the data of ≥ 2nd-line phase III trials on afatinib's efficacy. Erlotinib might be the standard control for trials focused on subsequent treatment after failure of chemotherapy in EGFR mutants.

### MTC of efficacy in patients with EGFR 19 Del / 21 L858R

According to MTC, 19 Del and 21 L858R got numerically greater survival benefit by TKI and chemotherapy, respectively. Moreover, rank probabilities also implied that the superiority of EGFR-TKI in OS in 19 Del patients could not be repeated in 21 L858R patients. Furthermore, chemotherapy revealed greater probability for better efficacy with regards to long-term survival compared with afatinib and erlotinib in 21 L858R patients. As a result, it confirmed that 19 Del was a distinct disease compared with 21 L858R. A well-designed clinical trial of chemotherapy vs. EGFR-TKI in 21 L858R patients should be performed separately in the future. Moreover, scientists should pay attention to the heterogeneity of genetic backgrounds between 19 Del and 21 L858R, which might give the original interpretation of the difference of the curative effect.

### MTC of adverse effects in EGFR mutants

MTC of toxicities showed that each TKI had its own merits and demerits. Afatinib, erlotinib and gefitinib had high, moderate and low risk of rash and diarrhea, respectively, while the occurrence of elevated LT was more common in gefitinib. Recommended EGFR-TKI should be suggested according to patients' tolerability and therapeutic efficacy in clinical practice. It is still unknown whether it is consistent between 19 Del and 21 L858R for dominant adverse effects of different TKIs. More efforts were encouraged to explore the above question in the future.

### Potential limitations

Nevertheless, there exist several limitations. Firstly, data of clinical outcomes were not available in some included studies which might influence statistically significances in comparison analyses. Secondly, we could not exclude the data of few non-classical EGFR mutants in the overall population which might have effects on the efficacy and toxicity of TKIs and cause potential bias. Future studies were warranted to further testify our results by replenishing unavailable data. MTC by adding the third generation EGFR-TKI (AZD9291 and CO1686) will be expected in a few years.

### Conclusion

Our study indicated a high efficacy-high toxicity pattern of afatinib, a high efficacy-moderate toxicity pattern of erlotinib and a medium efficacy-moderate toxicity pattern of gefitinib. Recommended EGFR-TKI should be suggested according to patients' tolerability and therapeutic efficacy in clinical practice. Moreover, the treatment for advanced EGFR-positive NSCLC might be different between 19 Del and 21 L858R.

## MATERIALS AND METHODS

### Study eligibility and identification

PubMed, Embase, Cochrane Central Register of Controlled Trials were searched respectively to find relevant articles using a combination of the terms “EGFR”, “mutation” “19 Del”, “21 L858R”, “Lung”, “NSCLC”, “TKI”, “gefitinib”, “erlotinib” and “afatinib”. We also reviewed abstract books and presentations of major recent meetings of ASCO, ESMO, ESMO-Asia and WCLC up to Dec. 2015 to ensure the latest research progress enrolled. Besides, all of the supplemental materials data from each trial were checked and extracted. The literature retrieval was carried out by three reviewers independently. Studies were included if they met the following criteria: (i) phase III randomized trials which reported advanced NSCLC EGFR mutants using specific EGFR-TKI treatment vs. chemotherapy or EGFR-TKI vs. another EGFR-TKI; (ii) trials might be performed in chemo-naïve patients or previously treated patients; (iii) EGFR-TKIs should be gefitinib, erlotinib or afatinib; (iv) EGFR-TKIs were not used as combined therapy or maintenance therapy; (v) at least one clinical outcome was available. Studies failing to meet the above inclusion criteria will be excluded from the network meta-analysis.

### Outcomes measures, data extraction and quality assessment

Therapeutic efficacy and toxicity were clinical outcomes including objective response rate (ORR), disease control rate (DCR), 1y-PFS rate, 1y-OS rate, 2y-OS rate and the rate of rash (grade 3-4 rash), diarrhea (grade 3-4 diarrhea), elevated liver transaminase (LT) (grade 3-4 elevated LT). The data on trial name, patient category, race, therapeutic regimens, EGFR mutation type and above clinical outcomes were extracted by two investigators independently. Two reviewers used the JADAD score to assess the quality of all included studies. [29] Discrepancies were discussed by all investigators to reach a consensus. All eligible studies were of high quality after the assessment.

### Statistical analyses

We conducted single-arm meta-analyses with a random effects model to synthesize rates of all the clinical outcomes in EGFR mutants, 19 Del patients and 21 L858R patients stratified by different EGFR-TKI treatments. The results were reported as pooled rates with the corresponding 95% confidence interval (CI). Statistical heterogeneity across studies was assessed with a forest plot and the inconsistency statistic (I^2^). All calculations were performed using R software, version 2.13.1.

After that, we built a random-effects network within a Bayesian framework using Markov chain Monte Carlo methods in ADDIS 1.15. [30, 31] We networked binary clinical outcomes within studies and specified the relations among the odds ratios (ORs) across studies to make comparisons of different treatments in EGFR mutants, 19 Del patients and 21 L858R patients as previously described. [17] P values less than 0.05 and 95% CIs were used to assess significance.

Moreover, the probability of the best regimen of each treatment in terms of efficacy and toxicity was also estimated by calculating the OR for each drug compared with an arbitrary common control group, and counting the proportion of iterations of the Markov chain in which each drug had the highest OR, the second highest, and so on. We also ranked the probability to be the best treatment among all the treatment regimens. Agents with greater value in the histogram were associated with greater probabilities for better efficacy or worse toxicity. The inconsistency within the network meta-analysis was evaluated by a variance calculation and a node-splitting analysis as previously reported. [31]

## SUPPLEMENTARY TABLES





## Abbreviation

EGFR-TKI: epidermal growth factor receptor-tyrosine kinase inhibitor
NSCLC: non-small cell lung cancer
19 Del EGFR: exon 19 deletions
21 L858R EGFR L858R: substitution in exon 21
PFS: progression-free survival
OS: overall survival
RCT: randomized controlled trial
MTC: multiple treatment comparisons
MTD: maximum-tolerated dose
ORR: objective response rate
DCR: disease control rate
LT: liver transaminase
OR: odds ratio
