# Supplementary material for: Optimized selection of three major EGFR-TKIs in advanced EGFR-positive non-small cell lung cancer: a network metaanalysis
Source: Oncotarget. 2016 Feb 25;7(15):20093–108. doi: 10.18632/oncotarget.7713 (PMC4990517; doi:10.18632/oncotarget.7713)
Supplement: Supplementary file 1 [file oncotarget-07-20093-s001.pdf]

SUPPLEMENTARY TABLES

Supplementary Table S1: Characteristics of treatment efficacy on eligible trials in EGFR mutants

Trial	Type	Race	Drug	EGFR mutants	ORR	DCR	1y-PFS	1y-OS	2y-OS
IPASS	CT naive	Asian	Gefitinib	132	94/131	121/131	46/132	106/132	57/132
			TC	129	61/127	113/127	10/129	97/129	58/129
NEJ002	CT naive	Asian	Gefitinib	114	84/113	102/113	50/114	97/114	66/114
			TC	114	35/107	91/107	5/110	99/114	61/114
WJTOG3405	CT naive	Asian	Gefitinib	86	36/58	54/58	34/86	74/86	55/86
			DP	86	19/59	46/59	10/86	81/86	60/86
OPTIMAL	CT naive	Asian	Erlotinib	82	68/82	79/82	47/82	69/82	41/82
			GC	72	26/72	59/72	1/72	57/72	42/72
First-SIGNAL	CT naive	Asian	Gefitinib	26	22/26	NA	9/26	19/26	16/26
			GC	16	6/16	NA	2/16	13/16	10/16
EURTAC	CT naive	Caucasian	Erlotinib	86	50/74	68/74	34/86	61/86	37/86
			GP/DP/ GC/DC	87	13/68	57/68	10/87	65/87	35/87
LUX-Lung 3	CT naive	Mixed	Afatinib	230	129/230	207/230	117/230	193/230	136/230
			AP	115	26/115	93/115	24/115	96/115	65/115
LUX-Lung 6	CT naive	Asian	Afatinib	242	162/233	224/233	136/242	194/242	109/242
			GP	122	28/99	93/99	7/122	95/122	57/122
ENSURE	CT naive	Asian	Erlotinib	110	69/110	98/110	35/110	91/110	60/110
			GP	107	36/107	82/107	13/107	88/107	58/107
LUX-Lung 7	CT naive	Mixed	Afatinib	160	112/155	146/155	76/160	NA	NA
			Gefitinib	159	89/156	139/156	65/159	NA	NA
V-15-32	Previously treated	Asian	Gefitinib	9	6/9	NA	NA	NA	NA
			DOC	11	5/11	NA	NA	NA	NA
INTEREST	Previously treated	Mixed	Gefitinib	19	8/19	NA	2/19	11/19	5/19
			DOC	19	4/19	NA	0/19	8/19	5/19
TITAN	Previously treated	Mixed	Erlotinib	7	NA	NA	6/7	5/7	3/7
			PEM/DOC	4	NA	NA	3/4	3/4	3/4
DELTA	Previously treated	Caucasian	Erlotinib	21	NA	NA	9/21	19/21	16/21
			DOC	30	NA	NA	7/30	24/30	19/30
WJOG 5108L	Previously treated	Asian	Gefitinib	161	99/155	133/155	64/161	NA	NA
			Erlotinib	150	83/143	127/143	62/150	NA	NA
CTONG 0901	Mixed(mainly on CT naïve)	Asian	Gefitinib	128	67/128	106/128	55/128	94/128	52/128
			Erlotinib	128	76/128	111/128	76/128	103/128	60/128
CTONG 0901	Previously treated	Asian	Gefitinib	44	NA	NA	14/44	26/44	14/44
			Erlotinib	47	NA	NA	27/47	36/47	23/47

Abbreviations: TC, carboplatin plus palitaxel; GP, cisplatin plus gemcitabine; DP, cisplatin plus docetaxel; DC, carboplatin plus docetaxel; DOC, docetaxel; GC, carboplatin plus gemcitabine; CT, chemotherapy; PEM, pemetrexed; AP, cisplatin plus pemetrexed; ORR, objective response rate; DCR, disease control rate; PFS, progression-free survival; OS, overall survival; NA, not available.

Supplementary Table S2: Characteristics of treatment efficacy on eligible trials in EGFR 19 Del patients

Trial	Type	Race	Drug	EGFR 19 Del mutants	ORR	DCR	1y-PFS	1y-OS	2y-OS
IPASS	CT naive	Asian	Gefitinib	66	56/66	NA	31/66	NA	NA
			TC	74	32/74	NA	8/74	NA	NA
WJTOG3405	CT naive	Asian	Gefitinib	50	NA	NA	17/50	NA	NA
			DP	37	NA	NA	3/37	NA	NA
OPTIMAL	CT naive	Asian	Erlotinib	57	36/46	45/46	27/57	NA	NA
			GC	58	17/41	41/41	6/58	NA	NA
EURTAC	CT naive	Caucasian	Erlotinib	113	73/113	104/113	NA	100/112	75/112
			GP/DP/ GC/DC	57	13/57	48/57	NA	46/57	27/57
LUX-Lung 3	CT naive	Mixed	Afatinib	124	97/124	117/124	NA	107/124	74/124
			AP	62	16/62	47/62	NA	47/62	28/62
LUX-Lung 6	CT naive	Asian	Afatinib	57	NA	NA	25/57	49/57	34/57
			GP	61	NA	NA	2/61	51/61	31/61
ENSURE	CT naive	Asian	Erlotinib	93	70/88	88/88	47/93	NA	NA
			GP	93	68/88	88/88	39/93	NA	NA
LUX-Lung 7	CT naive	Mixed	Afatinib	66	56/66	NA	31/66	NA	NA
			Gefitinib	74	32/74	NA	8/74	NA	NA

Abbreviations: 19 Del, exon 19 deletion; TC, carboplatin plus palitaxel; GP, cisplatin plus gemcitabine; DP, cisplatin plus docetaxel; DC, carboplatin plus docetaxel; GC, carboplatin plus gemcitabine; CT, chemotherapy; AP, cisplatin plus pemetrexed; ORR, objective response rate; DCR, disease control rate; PFS, progression-free survival; OS, overall survival; NA, not available.

Supplementary Table S3: Characteristics of treatment efficacy on eligible trials in EGFR 21 L858R patients

Trial	Type	Race	Drug	EGFR 21 L858R	ORR	DCR	1y-PFS	1y-OS	2y-OS
IPASS	CT naive	Asian	Gefitinib	64	39/64	NA	22/64	NA	NA
			TC	47	25/47	NA	4/47	NA	NA
WJTOG3405	CT naive	Asian	Gefitinib	36	NA	NA	16/36	NA	NA
			DP	49	NA	NA	8/49	NA	NA
OPTIMAL	CT naive	Asian	Erlotinib	29	16/22	21/22	8/29	NA	NA
			GC	29	12/22	22/22	3/29	NA	NA
EURTAC	CT naive	Caucasian	Erlotinib	91	51/91	85/91	NA	77/91	50/91
			GP/DP/ GC/DC	47	10/47	34/47	NA	43/47	31/47
LUX-Lung 3	CT naive	Mixed	Afatinib	92	48/92	82/92	NA	66/92	32/92
			AP	46	9/46	36/46	NA	38/46	26/46
LUX-Lung 6	CT naive	Asian	Afatinib	52	NA	NA	18/52	43/52	27/52
			GP	46	NA	NA	4/46	37/46	27/46
ENSURE	CT naive	Asian	Erlotinib	67	45/61	60/61	28/67	NA	NA
			GP	66	32/63	59/63	27/66	NA	NA
LUX-Lung 7	CT naive	Mixed	Afatinib	64	39/64	NA	22/64	NA	NA
			Gefitinib	47	25/47	NA	4/47	NA	NA

Abbreviations: 21 L858R, exon 21 L858R mutation; TC, carboplatin plus palitaxel; GP, cisplatin plus gemcitabine; DP, cisplatin plus docetaxel; DC, carboplatin plus docetaxel; GC, carboplatin plus gemcitabine; CT, chemotherapy; AP, cisplatin plus pemetrexed; ORR, objective response rate; DCR, disease control rate; PFS, progression-free survival; OS, overall survival; NA, not available.

Supplementary Table S4: Characteristics of treatment toxicities on eligible trials in EGFR mutants

Trial	Type	Race	Drug	EGFR mutants	Rash	Diarrhea	Elevated LT	Grade 3-4 Rash	Grade 3-4 Diarrhea	Grade 3-4 Elevated LT
NEJ002	CT naive	Asian	Gefitinib	114	81/114	39/114	63/114	6/114	1/114	30/114
			TC	114	25/113	7/113	37/113	3/113	0/113	1/113
WJTOG3405	CT naive	Asian	Gefitinib	86	74/87	47/87	61/87	2/87	1/87	24/87
			DP	86	7/88	35/88	35/88	0/88	0/88	2/88
OPTIMAL	CT naive	Asian	Erlotinib	82	61/83	21/83	31/83	2/83	1/83	3/83
			GC	72	14/72	4/72	24/72	0/72	0/72	1/72
EURTAC	CT naive	Caucasian	Erlotinib	86	67/84	48/84	5/84	11/84	5/84	2/84
			GP/DP/GC/DC	87	4/82	15/82	5/82	0/82	0/82	0/82
LUX-Lung 3	CT naive	Mixed	Afatinib	230	204/229	218/229	NA	37/229	33/229	NA
			AP	115	7/111	17/111	NA	0/111	0/111	NA
LUX-Lung 6	CT naive	Asian	Afatinib	242	193/239	211/239	48/239	35/239	13/239	4/239
			GP	122	10/113	12/113	18/113	0/113	0/113	3/113
ENSURE	CT naive	Asian	Erlotinib	110	78/110	50/110	13/110	7/110	NA	NA
			GP	107	11/104	9/104	2/104	1/104	NA	NA
LUX-Lung 7	CT naive	Mixed	Afatinib	160	142/160	144/160	15/160	15/160	19/160	0/160
			Gefitinib	159	129/159	97/159	38/159	5/159	2/159	12/159
CTONG 0901	Mixed (mainly on CT naïve) treated	Asian	Gefitinib	128	80/128	24/128	13/128	0/128	0/128	0/128
			Erlotinib	128	89/128	22/128	6/128	3/128	0/128	0/128

Abbreviations: TC, carboplatin plus palitaxel; GP, cisplatin plus gemcitabine; DP, cisplatin plus docetaxel; DC, carboplatin plus docetaxel; GC, carboplatin plus gemcitabine; CT, chemotherapy; AP, cisplatin plus pemetrexed; LT, liver transaminase, NA, not available.

Supplementary Table S5 Rank probabilities of each treatment for different outcomes based on network A and network B

See Supplementary File 1
