# Supplementary material for: Optimized selection of three major EGFR-TKIs in advanced EGFR-positive non-small cell lung cancer: a network metaanalysis
Source: Oncotarget. 2016 Feb 25;7(15):20093–108. doi: 10.18632/oncotarget.7713 (PMC4990517; doi:10.18632/oncotarget.7713)
Supplement: Supplementary file 2 [file oncotarget-07-20093-s002.docx]

**Table S5** Rank probabilities of each treatment for different outcomes based on network A and network B.

| **Drug** | **Rank 1** | **Rank 2** | **Rank 3** | **Rank 4** | **Rank 5** |
| --- | --- | --- | --- | --- | --- |
| **Total ORR (EGFR mutants)** |  |  |  |  |  |
| 1st-line Chemotherapy | 0 | 0 | 0 | 0.27 | 0.73 |
| Afatinib | **0.67** | 0.24 | 0.08 | 0.01 | 0 |
| After 1st-line Chemotherapy | 0.02 | 0.02 | 0.03 | 0.66 | 0.27 |
| Erlotinib | 0.28 | 0.52 | 0.18 | 0.02 | 0 |
| Gefitinib | 0.03 | 0.22 | 0.71 | 0.04 | 0 |
| **Total DCR (EGFR mutants)** |  |  |  |  |  |
| 1st-line Chemotherapy | 0 | 0 | 0.01 | 0.99 |  |
| Afatinib | 0.34 | 0.47 | 0.19 | 0 |  |
| Erlotinib | **0.63** | 0.32 | 0.05 | 0 |  |
| Gefitinib | 0.02 | 0.21 | 0.76 | 0.01 |  |
| **Total 1y-PFS (EGFR mutants)** |  |  |  |  |  |
| 1st-line Chemotherapy | 0 | 0 | 0 | 0.2 | 0.8 |
| Afatinib | **0.64** | 0.19 | 0.14 | 0.02 | 0 |
| After 1st-line Chemotherapy | 0.02 | 0.02 | 0.04 | 0.71 | 0.2 |
| Erlotinib | 0.21 | 0.38 | 0.39 | 0.02 | 0 |
| Gefitinib | 0.14 | 0.4 | 0.43 | 0.04 | 0 |
| **Total 1y-OS (EGFR mutants)** |  |  |  |  |  |
| 1st-line Chemotherapy | 0.11 | 0.36 | 0.35 | 0.15 | 0.03 |
| Afatinib | 0.39 | 0.24 | 0.18 | 0.15 | 0.04 |
| After 1st-line Chemotherapy | 0.04 | 0.04 | 0.03 | 0.07 | 0.82 |
| Erlotinib | **0.41** | 0.27 | 0.21 | 0.1 | 0.01 |
| Gefitinib | 0.05 | 0.09 | 0.23 | 0.53 | 0.1 |
| **Total 2y-OS (EGFR mutants)** |  |  |  |  |  |
| 1st-line Chemotherapy | 0.1 | 0.29 | 0.38 | 0.19 | 0.05 |
| Afatinib | **0.3** | 0.22 | 0.15 | 0.17 | 0.16 |
| After 1st-line Chemotherapy | 0.27 | 0.09 | 0.06 | 0.1 | 0.48 |
| Erlotinib | 0.27 | 0.28 | 0.22 | 0.16 | 0.06 |
| Gefitinib | 0.06 | 0.12 | 0.19 | 0.38 | 0.25 |
| **1st-line ORR (EGFR mutants)** |  |  |  |  |  |
| 1st-line Chemotherapy | 0 | 0 | 0 | 1 |  |
| Afatinib | **0.68** | 0.22 | 0.09 | 0 |  |
| Erlotinib | 0.27 | 0.54 | 0.19 | 0 |  |
| Gefitinib | 0.04 | 0.24 | 0.72 | 0 |  |
| **1st-line DCR (EGFR mutants)** | |  |  |  |  |
| 1st-line Chemotherapy | 0 | 0 | 0.02 | 0.98 |  |
| Afatinib | 0.35 | 0.47 | 0.17 | 0.01 |  |
| Erlotinib | **0.62** | 0.31 | 0.07 | 0 |  |
| Gefitinib | 0.03 | 0.22 | 0.74 | 0.01 |  |
| **1st-line 1y-PFS (EGFR mutants)** | |  |  |  |  |
| 1st-line Chemotherapy | 0 | 0 | 0 | 1 |  |
| Afatinib | **0.59** | 0.24 | 0.17 | 0 |  |
| Erlotinib | 0.27 | 0.36 | 0.37 | 0 |  |
| Gefitinib | 0.14 | 0.4 | 0.46 | 0 |  |
| **1st-line 1y-OS (EGFR mutants)** |  |  |  |  |  |
| 1st-line Chemotherapy | 0.11 | 0.42 | 0.39 | 0.08 |  |
| Afatinib | **0.46** | 0.22 | 0.17 | 0.15 |  |
| Erlotinib | 0.39 | 0.28 | 0.25 | 0.08 |  |
| Gefitinib | 0.04 | 0.08 | 0.19 | 0.69 |  |
| **1st-line 2y-OS (EGFR mutants)** |  |  |  |  |  |
| 1st-line Chemotherapy | 0.18 | 0.4 | 0.33 | 0.09 |  |
| Afatinib | 0.36 | 0.19 | 0.19 | 0.26 |  |
| Erlotinib | **0.37** | 0.24 | 0.22 | 0.17 |  |
| Gefitinib | 0.09 | 0.17 | 0.27 | 0.47 |  |
| **After 1st-line ORR (EGFR mutants)** | |  |  |  |  |
| After 1st-line Chemotherapy | 0.05 | 0.13 | 0.82 |  |  |
| Erlotinib | 0.28 | 0.56 | 0.16 |  |  |
| Gefitinib | **0.67** | 0.31 | 0.02 |  |  |
| **After 1st-line 1y-PFS (EGFR mutants)** | |  |  |  |  |
| After 1st-line Chemotherapy | 0.05 | 0.12 | 0.83 |  |  |
| Erlotinib | **0.69** | 0.28 | 0.03 |  |  |
| Gefitinib | 0.26 | 0.6 | 0.14 |  |  |
| **After 1st-line 1y-OS (EGFR mutants)** | |  |  |  |  |
| After 1st-line Chemotherapy | 0.05 | 0.26 | 0.7 |  |  |
| Erlotinib | **0.85** | 0.13 | 0.03 |  |  |
| Gefitinib | 0.11 | 0.62 | 0.28 |  |  |
| **After 1st-line 2y-OS (EGFR mutants)** | |  |  |  |  |
| After 1st-line Chemotherapy | 0.3 | 0.42 | 0.28 |  |  |
| Erlotinib | **0.6** | 0.31 | 0.09 |  |  |
| Gefitinib | 0.1 | 0.27 | 0.63 |  |  |
| **1st-line ORR (19 Del)** |  |  |  |  |  |
| 1st-line Chemotherapy | 0 | 0 | 0.04 | 0.96 |  |
| Afatinib | **0.44** | 0.42 | 0.14 | 0 |  |
| Erlotinib | 0.24 | 0.17 | 0.56 | 0.03 |  |
| Gefitinib | 0.32 | 0.41 | 0.26 | 0.01 |  |
| **1st-line DCR (19 Del)** |  |  |  |  |  |
| 1st-line Chemotherapy | 0.01 | 0.32 | 0.63 | 0.04 |  |
| Afatinib | 0.33 | 0.62 | 0.04 | 0 |  |
| Erlotinib | 0.01 | 0.03 | 0.18 | 0.77 |  |
| Gefitinib | **0.64** | 0.03 | 0.15 | 0.19 |  |
| **1st-line 1y-PFS (19 Del)** |  |  |  |  |  |
| 1st-line Chemotherapy | 0 | 0.01 | 0.05 | 0.93 |  |
| Afatinib | 0.37 | 0.36 | 0.22 | 0.05 |  |
| Erlotinib | **0.55** | 0.24 | 0.2 | 0.01 |  |
| Gefitinib | 0.08 | 0.38 | 0.53 | 0.01 |  |
| **1st-line 1y-OS (19 Del)** |  |  |  |  |  |
| 1st-line Chemotherapy | 0.02 | 0.41 | 0.58 |  |  |
| Afatinib | **0.75** | 0.22 | 0.03 |  |  |
| Erlotinib | 0.24 | 0.37 | 0.39 |  |  |
| **1st-line 2y-OS (19 Del)** |  |  |  |  |  |
| 1st-line Chemotherapy | 0.01 | 0.26 | 0.73 |  |  |
| Afatinib | **0.71** | 0.26 | 0.03 |  |  |
| Erlotinib | 0.28 | 0.47 | 0.24 |  |  |
| **1st-line ORR (21 L858R)** |  |  |  |  |  |
| 1st-line Chemotherapy | 0 | 0.03 | 0.29 | 0.69 |  |
| Afatinib | **0.77** | 0.22 | 0.01 | 0 |  |
| Erlotinib | 0.21 | 0.47 | 0.17 | 0.14 |  |
| Gefitinib | 0.02 | 0.28 | 0.53 | 0.17 |  |
| **1st-line DCR (21 L858R)** |  |  |  |  |  |
| 1st-line Chemotherapy | 0.03 | 0.56 | 0.4 | 0.01 |  |
| Afatinib | **0.85** | 0.14 | 0.01 | 0 |  |
| Erlotinib | 0.01 | 0.01 | 0.04 | 0.94 |  |
| Gefitinib | 0.11 | 0.28 | 0.56 | 0.05 |  |
| **1st-line 1y-PFS (21 L858R)** |  |  |  |  |  |
| 1st-line Chemotherapy | 0 | 0.01 | 0.05 | 0.94 |  |
| Afatinib | 0.37 | 0.29 | 0.29 | 0.04 |  |
| Erlotinib | **0.38** | 0.2 | 0.4 | 0.01 |  |
| Gefitinib | 0.24 | 0.49 | 0.26 | 0 |  |
| **1st-line 1y-OS (21 L858R)** |  |  |  |  |  |
| 1st-line Chemotherapy | 0.39 | 0.58 | 0.03 |  |  |
| Afatinib | 0.03 | 0.13 | 0.84 |  |  |
| Erlotinib | **0.58** | 0.29 | 0.13 |  |  |
| **1st-line 2y-OS (21 L858R)** |  |  |  |  |  |
| 1st-line Chemotherapy | **0.66** | 0.32 | 0.02 |  |  |
| Afatinib | 0.03 | 0.24 | 0.73 |  |  |
| Erlotinib | 0.31 | 0.44 | 0.25 |  |  |
| **1st-line Rash (EGFR mutants)** |  |  |  |  |  |
| 1st-line Chemotherapy | 0 | 0 | 0 | 1 |  |
| Afatinib | **0.81** | 0.14 | 0.05 | 0 |  |
| Erlotinib | 0.13 | 0.48 | 0.39 | 0 |  |
| Gefitinib | 0.06 | 0.39 | 0.55 | 0 |  |
| **1st-line Diarrhea (EGFR mutants)** | |  |  |  |  |
| 1st-line Chemotherapy | 0 | 0 | 0 | 1 |  |
| Afatinib | **1** | 0 | 0 | 0 |  |
| Erlotinib | 0 | 0.6 | 0.39 | 0 |  |
| Gefitinib | 0 | 0.4 | 0.6 | 0 |  |
| **1st-line Elevated LT (EGFR mutants)** | |  |  |  |  |
| 1st-line Chemotherapy | 0 | 0.03 | 0.31 | 0.65 |  |
| Afatinib | 0.02 | 0.23 | 0.46 | 0.29 |  |
| Erlotinib | 0.05 | 0.68 | 0.22 | 0.05 |  |
| Gefitinib | **0.93** | 0.06 | 0.01 | 0 |  |
| **1st-line Grade 3-4 Rash (EGFR mutants)** | |  |  |  |  |
| 1st-line Chemotherapy | 0 | 0 | 0.06 | 0.94 |  |
| Afatinib | **0.5** | 0.45 | 0.04 | 0 |  |
| Erlotinib | 0.49 | 0.42 | 0.09 | 0 |  |
| Gefitinib | 0.01 | 0.12 | 0.81 | 0.06 |  |
| **1st-line Grade 3-4 Diarrhea (EGFR mutants)** | |  |  |  |  |
| 1st-line Chemotherapy | 0 | 0 | 0 | 1 |  |
| Afatinib | 0.23 | 0.69 | 0.08 | 0 |  |
| Erlotinib | **0.74** | 0.08 | 0.18 | 0 |  |
| Gefitinib | 0.03 | 0.23 | 0.74 | 0 |  |
| **1st-line Grade 3-4 Elevated LT (EGFR mutants)** | |  |  |  |  |
| 1st-line Chemotherapy | 0 | 0.05 | 0.7 | 0.25 |  |
| Afatinib | 0 | 0.06 | 0.22 | 0.72 |  |
| Erlotinib | 0.24 | 0.66 | 0.07 | 0.03 |  |
| Gefitinib | **0.76** | 0.23 | 0.01 | 0 |  |

Abbreviations:19 Del,exon 19 deletion; 21 L858R, exon 21 L858R mutation; ORR, objective response rate; DCR, disease control rate; PFS, progression-free survival; OS, overall survival; LT, liver transaminase.
